# Nutritional risks in children with food allergy

**DOI:** 10.55730/1300-0144.5648

**Published:** 2023-04-04

**Authors:** Zeynep PARLAK, Deniz İLGÜN GÜREL, Özge SOYER, Bülent Enis ŞEKEREL, Ümit Murat ŞAHİNER

**Affiliations:** 1 Department of Pediatric Allergy and Asthma, Faculty of Medicine, Hacettepe University, Ankara, Turkiye; 2 Department of Nutrition and Dietetics, Hacettepe İhsan Doğramacı Children’s Hospital, Hacettepe University, Ankara, Turkiye

**Keywords:** Food allergy, nutritional risk, cow’s milk allergy

## Abstract

Food allergies (FA) are a growing problem in the pediatric population and clinical features differ according to the underlying immunological mechanisms. While the primary management strategy is to eliminate the culprit food from the diet, assessment of the potential nutritional risks of elimination is also an integral part of management. In cases that do not improve over time; if you have basic food allergies and multiple food allergies, this can also lead to negative nutritional consequences. The contribution of basic nutrients, economical and easily accessible foods to the diet, is critical and has an important place in meeting the daily adequate intake of many nutrients. In the presence of food allergy, it is necessary to meet the vitamins and minerals that cannot be obtained from allergic foods, with alternative sources or supplements. For example, insufficient calcium intake in cow’s milk allergy (CMA), the most common FA in early childhood, is very likely if an alternative supplement has not been introduced. In the management of CMA, choosing the appropriate formula and/or supplement for the clinical characteristics of children, when necessary, has an important place. In conclusion, nutritional risk assessment of children with FA requires a comprehensive, detailed, and multidisciplinary approach.

## 1. Introduction

Food allergies (FA) continue to be an increasing problem in pediatric practice. Although allergenic foods are diverse and their prevalence differs in populations, a small number of foods are responsible for most allergic reactions in young children. In addition to milk, eggs, wheat, soy, peanuts, tree nuts, fish, shellfish, and sesame are the most common allergens [1–4].

Effective management of FA requires a comprehensive, detailed, and multidisciplinary approach starting from its diagnosis. Clinical management of FA is based on two main strategies which include treatment of acute reactions and avoidance of the culprit food [5]. Nutritional management encompasses not only the elimination of responsible foods but also the prevention of nutritional deficiencies. However, many components are included to ensure successful avoidance (i.e. label reading; possible contaminations in restaurants and school cafeterias) and the degree of nutrient elimination necessitates individually a broad perspective of nutritional management [6].

The clinical presentations of adverse reactions to food are diverse, and differences in emerging symptoms and clinical management are other factors that diversify nutritional risks. Therefore, it is important to know the possible mechanisms associated with the clinical presentation when determining potential nutritional risks [7,8].

The purpose of this review is to identify potential nutrient deficiencies in the nutritional management of FA in children.

## 2. Nutritional risks in different forms of food allergy

Food allergy includes reactions to ingested antigens through various immunological mechanisms and is classified according to the involvement of IgE-mediated and/or other immune responses [9]. The strict elimination of the responsible food from the diet means the removal of all forms of the food from the diet and may compromise adequate and balanced nutrition. However, another important point to be considered is the type of FA. Symptoms are very diverse and variable in severity. Therefore, it is important to know the accompanying comorbidities that increase nutritional risks.

Symptoms in IgE-mediated FA are usually characterized by their rapid onset after exposure to the allergen. Signs and symptoms may be mild or localized, or may involve different organ systems simultaneously in anaphylaxis [9,10]. In IgE-mediated FA, reactions can occur with a wide variety of foods in different age groups, but the most common causes of anaphylaxis are peanut and tree nuts and shellfish. In early childhood, cow’s milk and eggs are the major triggers [11–13]. However, as the age progresses, cultural differences become more important. Unlike the USA, where peanuts are the most common food allergen by age; the most common food allergens in older children in Turkey are tree nuts and sesame [14]. In a study examining the etiology of food-induced anaphylaxis in our country; cow’s milk, tree nuts, and eggs were found to be the most common triggers in infants and tree nuts were the most common triggers for toddlers and preschoolers [15]. Atopic dermatitis (AD) is a chronic inflammatory skin disease which, affects 10% to 20% of the pediatric population, and approximately one-third of children with severe AD have IgE-mediated clinical reactivity to food proteins [16]. Cow’s milk and egg are the most common causes of FA in children with AD [17]. It has been reported that growth is affected especially in patients with food allergies accompanied by eczema [18,19]. In addition to the elimination diet, if AD is not treated, deterioration in sleep quality, increase in energy and protein requirements may lead to an increase in nutritional risk [20].

Food Protein-Induced Allergic Proctocolitis (FPIAP), most commonly caused by cow’s milk, occurs in early infancy. The responsible food may need to be excluded from the maternal diet. Since it occurs in early childhood and the most common cause is milk, therapeutic formulas may be needed if the breastfeeding is not sufficient and/or if there is any cause of contraindication for breastfeeding [21]. Nutritional risk is minimal in cases of proctocolitis, as tolerance usually develops in the first year of life. Although cow’s milk is the most common trigger in children with FPIAP, in some cases, other foods may accompany cow’s milk. In the study in which FPIAP phenotypes were examined, cow’s milk was accompanied by other foods in 36.7% of the participants [22]. In particular, iron stores may be reduced or depleted in long-term symptomatic cases that require multiple elimination, so these parameters should be closely monitored [23]. In addition, although the nutritional risk is relatively low, delaying complementary feeding and expanding elimination unnecessarily, increase the potential nutritional risks [24,25].

Food protein-induced enterocolitis syndrome (FPIES) is non-IgE-mediated FA usually characterized by recurrent vomiting in infants. The onset of FPIES in the breastfed infant may be delayed, usually because it is not triggered by food proteins that pass-through the breast milk [26,27]. In these cases, the initial reaction may occur after consumption of formula or during the complementary feeding period in which solid foods are included in the diet. Between 65% to 80% of children with FPIES are sensitive to a single food, and cow’s milk is the most common offending food. Children with cow’s milk and soy induced FPIES may have reactions to both foods in 30%–40% of cases. These children may also have an increased likelihood of reacting to a solid food, most commonly rice or oat and egg [26,28]. In our pediatric allergy department, the most common oral food challenges performed in patients with FPIES were with egg, fish and cow’s milk [29]. FPIES has a wide range of clinical characteristics, including acute or chronic, and mild, moderate, or severe symptoms. For this reason, children with FPIES are at increased nutritional risk, such as poor feeding skill acquisition and inadequate nutrient intake due to delayed solid foods and long-term elimination [25,27].

Eosinophilic esophagitis (EoE) is a disease of increasing recognition and prevalence, clinically characterized by symptoms of esophageal dysfunction and histologically eosinophil-dominated inflammation. Symptoms such as nausea, vomiting, abdominal pain, dysphagia, dysphagia-related delayed growth and feeding difficulties can be observed. In recent years, clinical studies on the treatment of EoE demonstrated the benefit of topical corticosteroids, proton pump inhibitors, limited elimination diets, and biologics for remission of EoE [30]. Elimination of food antigens has become part of the treatment, due to data that food allergens play a role in the pathogenesis of the disease [31,32]. There is no test to definitively identify the triggering food antigen consequently leading to use of empirical elimination diets or elemental diets as dietary therapy. The elemental diet consists of consumption of an amino acid-based formula which results in remission of the disease in the children with EoE [33]. However, elemental diet practice in children requires attention and close follow-up. The amino acid-based formula to be recommended should be determined by considering the energy and nutritional requirements. Elemental diet in children; despite remission, can adversely affect energy, nutrient intake, food acceptance, taste preference and oral motor skills [34]. The studies evaluating the effects of 6 food elimination diets (milk, wheat, egg, soy, peanut/tree nuts, and fish/ shellfish), and 4 food elimination diets (milk, egg, wheat, and legumes) reported more than 70% histological response. The most common implicated foods were cow’s milk and wheat as confirmed by control esophageal biopsies [35–37].

When evaluating nutritional status, clinical characteristics and FA symptom severity should also be considered. Children with untreated or uncontrolled food allergies are at greater risk of nutrient deficiencies.

## 3. Macro and micronutrient deficiencies in children with food allergy

Nutrients are classified as macronutrients consisting of carbohydrates, proteins and fats, and micronutrients consisting of vitamins, minerals, and trace elements. The amount of nutrients required for normal physiological functioning may vary according to age, gender, and presence of chronic diseases. The unmet requirements of the body in terms of these nutrients, result in deficiencies. This imbalance can also result from impaired nutrient absorption or increased nutrient needs due to different factors. Nutrient deficiencies can lead to impaired growth, depletion of body stores and tissue concentrations. As a result, it disrupts the metabolic pathways leading to clinical symptoms [38,39].

**Figure 1:**
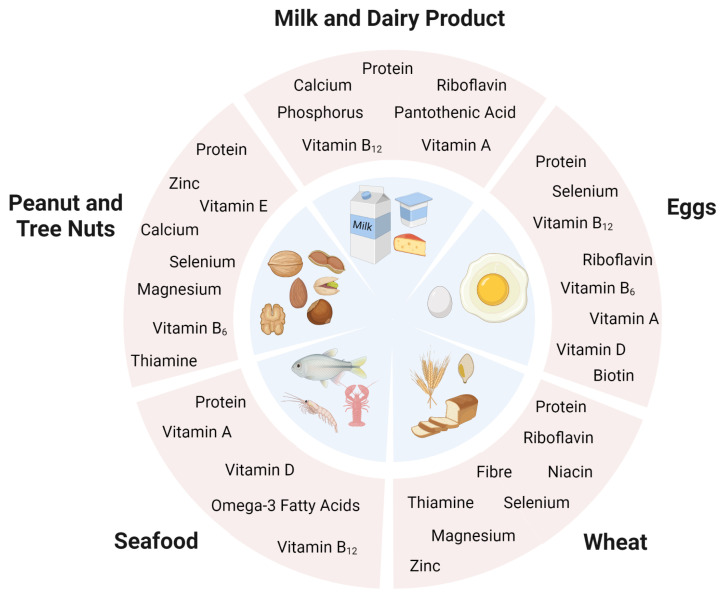
The major macronutrient and micronutrient contribution of common allergenic foods to the diet.

Food allergy in children mostly develops within the first 2 years of life, a period critical for growth and development. The most common allergenic foods provide important macronutrients and micronutrients to a developing child’s diet. The major macronutrient and micronutrient contribution of common allergenic foods to the diet are shown in Figure 1.

### 3.1. Macronutrients

A healthy diet is based on a variety of foods, and it is essential to include nutrients from the main food groups in the daily diet in different portion sizes. In addition, amount and frequency of consumption of food groups vary according to societies. Protein, carbohydrates, and fats are energy-providing macronutrients, and an energy deficit is a primary nutrient deficiency. However, the distribution of macronutrients is also important. The nutrient contribution of common allergenic foods to the diet is variable. Cow’s milk and dairy products make an important contribution to the early childhood diet and being responsible for most of the allergic reactions during this period has converted it to become the focus of studies.

Children with cow’s milk elimination from diet had lesser protein and lipid intake compared to the control group, which resulted in lower energy levels [40]. In another study, the food intake of children older than two years on a diet eliminating cow’s milk was evaluated, and the average energy intake, the percentage of energy from protein and fat was found to be significantly lower than the control group [41]. There are also data reporting that energy level and macronutrient intake is similar to the healthy group and is not affected by the number of foods eliminated [42].

In a study by Berry et al. energy, protein and fat intake were lower in children with cow’s milk allergy (CMA) accompanied by wheat allergy. However the difference was not significant compared to those with only milk allergy [43]. In many studies, the macronutrient intake of children with food allergies was mostly calculated on nutritional diaries. However, growth is an important long-term indicator of adequate energy and protein intake, with low energy intake is associated with poor growth in children.

Isolauri et al. [44], reported decreased growth in young children with CMA compared to healthy children and 6% of the patients had low serum albumin and 8% had low serum phospholipid docosahexaenoic acid levels. Christie et al. [45] reported that children who eliminated two or more foods were of lower height for their age than those who eliminated only one food. In the study of Flammarion et al. [42], although energy and protein intakes were similar, the weight for age and height-for-age z-scores of children with food allergies were found to be lower than those of the controls. In the same study, children who eliminated 3 or more food were shorter than those who eliminated 2 or fewer foods. Meyer et al. [46], reported that elimination of 3 or more foods affected the weight-for-age z-score. These data suggest an increased risk in growth, especially in children with multiple food allergies. Elimination of foods that contribute significantly to nutrition, as well as accompanying comorbidities, are also thought to affect growth. Dhar et al. [47] reported growth retardation in children with AD, increasing with surface area of involvement and severity of AD. In a population-based cohort study examining the relationship between IgE-mediated FA, eczema and anthropometric measures; children with eczema and food allergies were reported to have shorter stature and lower weight during early childhood than children without any condition [19]. In another study, the concomitant presence of IgE-mediated allergy, particularly milk allergy, has been reported to significantly impair growth in children with AD [18].

Vieira et al. [48] demonstrated that in approximately one-fourth of the study population with CMA and the concomitant major gastrointestinal symptoms, growth was stunted. In another study in children with food protein induced gastrointestinal allergies, the rate of stunting was 9% [49]. This difference in results is thought to be due to different populations and possible dietary intake. Eosinophilic esophagitis has serious nutritional risks due to its strict and extensive nutrient elimination and clinical characteristics. The effect of dietary therapy (avoiding the 6 most common allergenic foods, as well as avoiding those that reveal positive skin tests and using special formulas instead of dairy) in children with EoE was examined and no significant negative impact was reported [50].

Although clinical features, age groups and eliminated foods are different in these studies, they reveal that growth follow-up has an important place in the nutritional management of children with food allergies. However, the possible differences in the nutritional habits of the different societies in which the studies were conducted should not be overlooked. In our country, besides milk and eggs, wheat is a basic food that is consumed quite frequently, especially in the form of bread. Therefore, the possible nutritional risks of wheat allergy may be exacerbated.

### 3.2. Micronutrients

#### 3.2.1. Vitamins and minerals

Providing the vitamins and minerals necessary for the optimal continuity of growth and development in early childhood is possible with a nutritional model with high nutritional value [51]. Foods such as milk, eggs, and nuts, which are often the cause of food allergies in children, are nutrients that contribute significantly to vitamin and mineral intake. Therefore, children with food allergies are at risk of vitamin and mineral deficiencies [24]. Undernutrition for vitamins and minerals progresses in stages and may result from inadequate intake of nutrients, malabsorption, impaired metabolism, nutrient loss, or increased nutritional requirements. In the first stage, nutrient levels in the blood and tissues change, followed by intracellular changes in biochemical functions and structure leading to signs and ultimately symptoms [52].

Low or insufficient intake of calcium, phosphorus, zinc, vitamin D, riboflavin and niacin has been reported in children with FA, by different studies [40, 41, 45, 53]. Flammarion et al. demonstrated higher dietary intake of vitamin A and vitamin E in children with food allergies compared to healthy controls, and this result was explained by the vegetable oils added to the diet in the FA group [42]. In studies evaluating the dietary intake of vitamins and minerals in children with food allergies, nutrient intake was calculated based on dietary records or food consumption frequencies. In these studies, nutrient intakes of the FA children were compared with that of the healthy controls or with the percentages of recommended daily intake levels for each nutrient according to age groups. While evaluating the results of dietary intake, it should be considered that the databases in which the vitamin and mineral content of foods are calculated, and the recommended dietary intakes may vary in different countries.

Various micronutrient deficiencies have been noticed in children with food allergies. A prospective, multicenter intervention study found zinc deficiency in patients who did not receive dietary counseling [53]. In a study in which B12, iron and zinc deficiencies were reported in children with CMA, the B12 levels of those feeding on hypoallergenic formula were significantly higher than those of breastfeeding [54]. Meyer et al. evaluated the effect of the use of hypoallergenic formula on micronutrient intake in patients with food protein-induced gastrointestinal allergy, reporting that it positively affected dietary intake for most micronutrients [55]. Iron deficiency is the most common micronutrient deficiency worldwide also in young children, regardless of FA [56]. Therefore, iron deficiency should not be associated only with food elimination. The occurrence and severity of micronutrient deficiencies can be affected by clinical features such as malabsorption and inflammation. In a study comparing trace element levels in children diagnosed with AD versus healthy children, serum magnesium and erythrocyte zinc levels were found to be lower regardless of the severity of AD and these outcomes have been associated with chronic inflammation [57].

**Figure 2:**
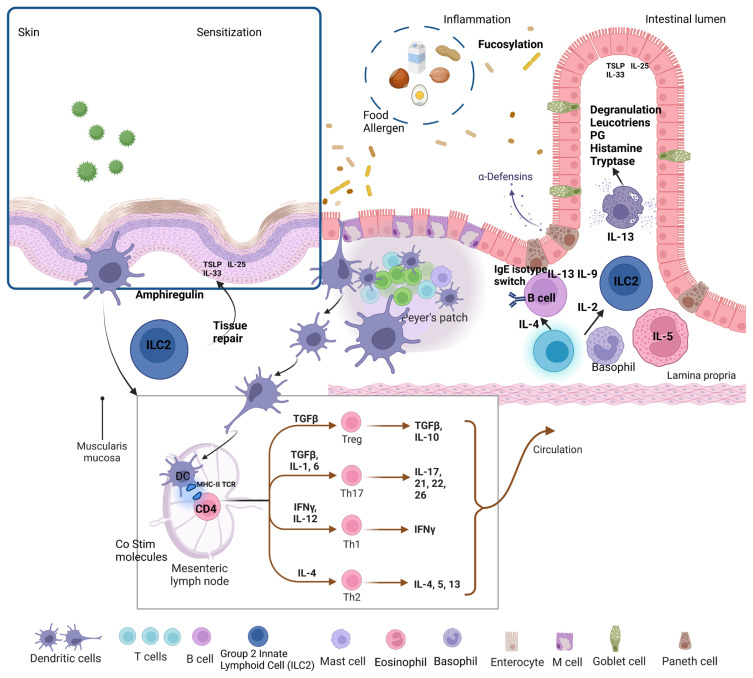
Dysregulation of the gut-skin axis in atopic dermatitis.

Cutaneous inflammation in AD sends signals to B cells and promotes the production of antigen-specific IgE, resulting in the generation of IL-4 and IL-13 from active TH2 cells, leading to the formation of TH2 cell-mediated pathways. Whether the eczematous potency can be induced by food intake is still debated. The cutaneous inflammatory infiltrate in the eczematous skin of patients with AD consists mainly of CD4+ T cells [58]. Allergens stimulate the production of thymic stromal lymphopoietin (TSLP), IL-33 and IL-25 in the skin keratinocytes; these alarmins induce the activation of group 2 innate lymphoid cells (ILC2s) and dendritic cells (DCs). Migration of DCs to lymph nodes triggers proliferation of Th2 effector and memory cells. It is hypothesized that upon ingestion of food to which the individual is sensitized, these Th2 cells migrate to the gut and communicate with ILC2s, which in turn produce IL-13. Intestinal epithelial cells also produce IL-33 and IL-25, which further stimulate ILC2s [59]. In addition, the immunogenicity of pathogens and food antigens is reduced by luminal and brush-edged enzymes, bile salts, and excessive pH values in the gastrointestinal tract. These functional barrier mechanisms include defense against foreign antigens, components of intestinal innate immunity (polymorphonuclear neutrophils, macrophages, natural killer cells, epithelial cells, and Toll-like receptors) and adaptive immune response (intraepithelial and lamina propria lymphocytes, Peyer’s patches, glands, IgA and cytokines) [60]. The immaturity of various components of the gut barrier and gastrointestinal immunity may explain the high rate of food allergies in infants and young children [61]. Dysregulation of the gut-skin axis in AD is shown in Figure 2. It has been reported that children with food allergies have lower serum selenium and zinc levels compared to healthy children, and the mean serum selenium and zinc levels increase after the elimination diet [62]. In another study, more than 15% of children with non-IgE mediated gastrointestinal food allergies, the majority of whom had multiple food elimination, had low levels of vitamin A, zinc, selenium, and copper [63]. These results suggest that children with food allergies had a weakened antioxidative barrier and trace elements should be monitored in children with food allergies.

Thomassen et al. studied iodine levels in children with CMA by comparing urinary iodine concentrations with WHO cut-off values for iodine deficiency and in one-third of the children urinary iodine concentrations were low indicating iodine deficiency [64]. The highest risk of deficiency was found in breastfed infants (58%). However, no correlation was indicated between growth and iodine deficiency.

#### 3.2.2. Calcium and vitamin D

Rickets is a disease of childhood caused by failure or delay in endochondral calcification at the growth plates of long bones, leading to skeletal deformities, impaired growth, and other clinical features. It is classified according to the pathogenetic mechanisms involved in the formation of the disease. While vitamin D deficiency is the most common cause of rickets, nutritional rickets can also result from a lack of dietary vitamin D, calcium, or phosphorus [65,66]. Vitamin D is a fat-soluble, secosteroid prohormone with the classical function being calcium and phosphate homeostasis, when 7-dehydrocholesterol is exposed to ultraviolet B (UVB) irradiation, causes endogenous synthesis endogenously of vitamin D in the skin. It can also be partially ingested from dietary sources. A certain number of foods naturally contain vitamin D but if vitamin D is not fortified, it is expected that dietary vitamin D will be low. The serum concentration of 25(OH)D is currently considered the best marker of vitamin D status. On the other hand the serum calcium concentration is a poor parameter by itself to assess the calcium status in the body [67, 68]. For many societies, the most important dietary sources of calcium are milk and dairy products. Calcium contents of some foods are shown in Table 1. The contribution of dairy products to total calcium intake is estimated to be more than 50% in the USA, Germany, and England, while comprising more than 70% in the Netherlands [68]. For this reason, insufficient consumption of milk and dairy products as a consequence of a medical condition such as CMA may cause the risk of low calcium intake.

It has long been known that children with cow’s milk protein allergy and multiple food allergies have a lower calcium intake in their daily diet [45]. In another study evaluating the nutritional status of children in whom milk and dairy products were eliminated; calcium and phosphorus intake were found to be significantly lower [40]. In addition, many cases of nutritional rickets have been reported with CMA where vitamin D and calcium are not adequately supplemented [69–72]. Also, there are studies evaluating bone mineral status in children with cow’s milk protein allergy. Jensen et al. [73] reported that children who followed an elimination diet due to a diagnosis of CMA for more than four years had lower bone mineral content and density, and their bone age was delayed by 1.4 years. In this study, calcium consumption, calculated from the food intake of children with CMA, comprised only 25% of the recommended value. Additionally, inhaled corticosteroid treatment might also influence the low bone mineral density of the participants. Mailhot et al. [74] notified lower bone mass and calcium intake in prepubertal children with persistent milk allergy. However, no correlation was found between calcium intake, vitamin D levels and bone mass. It was assumed that this might be due to the use of steroids, which negatively affected calcium absorption in the patient group, and the inclusion of other beneficial minerals for bone health present in milk and dairy products in the healthy group. In another study concerning the bone mineral density of IgE-mediated milk allergy, desensitized and healthy children, densitometric measurements of the hip, femoral neck, and lumbar spine of patients with milk allergy were significantly lower than those in the healthy group. However, no significant difference was displayed between allergic patients and healthy controls after desensitization [75].

There are many factors that affect nutritional management in children with food allergies. However, it is important to add alternative sources of eliminated foods to the diet, considering the nutrient needs according to age. In addition, detection of possible nutrient deficiencies is important to identify patients who need supplementation. In Table 2, alternative sources of nutrients in common allergenic foods, adequate intake levels and biochemical markers used to measure nutrient levels in blood and tissues are shown.

## 4. Nutrient contribution of milk substitutes

Cow’s milk allergy is a very common FA in infants [76]. If breast milk is unavailable or inadequate dietary intervention should include a hypoallergenic formula. The first choice can be hypoallergenic intensive hydrolyzed cow’s milk formulas (eHF). Amino acid-based formulas (AAF) are the other option for patients with severe symptoms or who are unresponsive to eHF [77,78].

Rice is a nonallergenic cereal. Non-IgE-mediated enterocolitis associated with dietary rice protein intake may be caused less frequently than milk and soy protein [79,80]. Hydrolyzed rice-based formulas (HRFs) may be an option for infants with CMA and infants with severe forms of CMA who cannot tolerate or dislike eHF/AAF [81,82]. Essential amino acid ingredient of rice is less than human milk content. These amino acids are tryptophan, 17 mg vs. 9 mg, threonine, 37 mg versus 44 mg; and lysine, 36 mg vs. 67 mg. Supplementation of the amino acids are L-threonine, L-lysine, and L-tryptophan is necessary and an infant’s amino acid requirements cannot be fully met when these amino acids are deficient and [81]. Looking at the study results, they evaluated whether infants with CMA fed HRF allowed growth and adequate metabolic balance. No significant difference was found between head circumference, height, weight, and BMI for 4 months in infants fed regular infant formula and hydrolyzed rice-based formula [83]. In the study of Reche et al., it was observed that most infants (≥%90) with cow’s milk protein allergy tolerate rice protein-based formula well [84]. At the end of this study, they concluded that HRF may be a suitable nutritional alternative for infants with CMA.

Developmental milestones (motor, mental, and language) of breastfed (BF), soy protein-based milk-fed or milk-based formula-fed (MF) infants were characterized and total scores on the developmental test were within identified normal ranges and showed that the no significant difference was observed between MF and soy-based formula (SF) groups. Additionally, this study showed that BF infants had a slight advantage in cognitive development compared to formula-fed infants [85]. Adequate and properly administered complementary foods ensure the proper growth and development of children [86].

Before the discovery of therapeutic formulas based on cow’s milk protein hydrolysates, soy formula was the only dietary product available for feeding infants with cow’s milk protein allergy. However, soy protein is also a common allergen. Among infants with CMA fed soy protein-based formulas, approximately 30% to 50% have been reported to have concomitant soy protein allergy and a higher frequency of non-IgE-mediated enterocolitis-enteropathy syndrome has been reported [87]. Besides, approximately 14% of infants with CMA may also have a reaction to soy-based infant formulas [88].

Soy formula includes noteworthy amounts of phytoestrogens (like isoflavones) and should be kept off during the first six months of life [89]. In some countries, it is suggested as a second-line treatment for CMA [90].

Soy protein isolate contains about 1% to 2% phytates, which can impair the absorption of minerals and trace elements. Phytate has an adverse effect on intestinal zinc and iron absorption in experimental animals and human [91]. Persistence of thyroid insufficiency despite high doses of levothyroxine has also been observed in infants with congenital hypothyroidism who have recently been fed soy protein formulas [92].

The use of soy formulas may play a role in the etiology of peanut allergy. Evaluating data from the Avon longitudinal study, a cohort study of a large population of preschoolers, Lack et al., showed that soy milk or soy infant formula was independently associated with peanut allergy intake in the first 2 years of life (CI 2.6; %95 GA 1.4–5.0), which suggested the possibility of cross-sensitization via common epitopes. Soy protein fractions have been shown to be homologous to major peanut proteins [93].

Plant-based beverages (PBB) are mainly industrial products produced from legumes, tree nut, seeds, and cereals through different processes. Based on usage similarity with cow milk, they have hit the marketplace as “milk alternative” but according to their nutritional aspect they have a different content from cow’s milk. For children above 2 years old, alternatively, calcium enriched PBB (e.g., soya milk, oat milk, almond milk, coconut milk, rice milk) can also be used [94,95]. Nutritional composition of PBBs varies according to the plant source it is being obtained from (e.g., soy, almond, oat, cashew, potato, hazelnut), processing techniques and whether it is being or not enriched with vitamins and/or minerals [96]. Data on the consumption of PBBs in children are scarce and their role in meeting children nutritional need is still unclear [97,98]. Range of nutrients content of milk substitutes in our country is shown Table 3.

## 5. Conclusion

Elimination of the responsible food in food allergies is the most important part of FA management and especially children with multiple food allergies are at risk of macro and micronutrient deficiencies and insufficient growth. The clinical manifestations of reactions to food are diverse, and differences in symptoms and clinical management are another important factor that can lead to nutritional deficiencies. Therefore, diet in a child with FA requires follow-ups to ensure that not only the allergic symptoms are treated, but also to ensure optimal growth requirements are met. The use of suitable substitutes in CMA, which is very common in early childhood, is important in terms of its contribution to nutrition as well as the management of FA. For all these reasons nutritional management of FA requires, after a comprehensive risk assessment, establishing an adequate and balanced diet necessary for optimal growth and development, and monitoring dietary adherence.

## Figures and Tables

**Table 1:** Mean calcium content of foods.

Calcium content of some foods (100 g/mg)*			
**Foods**	mean (min-max)	**Foods**	mean (min-max)
**Cow’s milk**	98 (70–116)	**Egg**	52 (43–61)
**Yogurt**	132 (132–132)	**Black cabbage**	569 (353–1030)
**Feta cheese**	422 (422–422)	**Grape leaf**	1421 (1421–1421)
**Sesame**	693 (197–1133)	**Leek**	50 (29–74)
**Tahini**	167 (167–167)	**Spinach**	143 (40–239)
**Almond**	247 (238–267)	**Swiss chard**	110 (79–181)
**Hazelnut**	111 (111–111)	**Orange**	63 (38–88)
**Pistachio**	117 (72–140)	**Kiwi**	36 (28–43)
**Walnut**	103 (90–124)	**Fig (dried)**	140 (97–184)
**Sunflower seed**	66 (66–66)	**Apricot (dried)**	62 (51–76)
**Lentil**	26 (26–26)	**Grape molasses**	32 (30–35)
**Chickpea (boiled)**	62 (62–62)	**Mulberry molasses**	108 (108–108)

* National Food Composition Database (TÜRKOMP)

**Table 2:** Alternative sources of nutrients and recommended adequate intake levels in children with food allergy.

Nutrient	Common allergen sources for nutrients	Substitute foods/alternative sources of similar nutrients	Recommended adequate intakes for nutrients by Age*	Assessment of nutrients status
**Protein**	Milk and dairy products, seafood, eggs, peanuts and tree nuts, soy	Meat, poultry, legumes, therapeutic formula	Recommended adequate intake for protein is calculated based on body weight	Serum albumin level [99] Serum prealbumin, and Total protein level [83]
**Calcium**	Milk and dairy products, tree nuts, tahini, sesame seeds, enriched soy products, fish bones	Fortified alternative beverages, dark green leafy vegetables, therapeutic formula	2–3 years: 450 mg 4–10 years: 800 mg 9–13 years: 1150 mg	Serum calcium level [100]
**Zinc**	Shellfish, tree nuts, sesame, tahini, whole wheat, soy	Beef, poultry, fortified cereals, legumes, leafy and root vegetables	2–3 years: 4.3 μg 4–6 years: 5.5 μg 7–10 years: 7.4 μg 11–13 years: 10.7 μg	Serum zinc level [101]
**Selenium**	Peanut and tree nuts, fish, eggs, whole wheat	Meat, poultry, sunflower seeds, pumpkin seeds	2–3 years: 15 μg 4–6 years: 20 μg 7–10 years: 35 μg 11–13 years: 55 μg	Serum selenium level [101]
**Vitamin A**	Fortified milk and milk products, seafood, egg yolk	Dark green leafy vegetables, deep orange fruits and vegetables, plant oils	2–3 years: 250 μg 4–6 years: 300 μg 7–10 years: 400 μg 11–13 years: 600 μg	Serum vitamin A level [101], serum β-carotene level and serum retinol level [102, 103]
**Vitamin D**	Fortified milk and milk products, fortified wheat-based cereals, fatty fish, egg yolk	Fortified cereals, fortified alternative beverages	2–13 years: 600 IU	25-OH vitamin D serum level [104]
**Vitamin E**	Peanuts, tree nuts	Plant oils (e.g., sunflower, olive, canola), sunflower seeds	2 years: 6 mg 3–9 years: 9 mg 9–13 years: 11 mg (female)/13 mg (male)	α-tocopherol level [102, 103]
**Vitamin B12**	Milk and dairy products, eggs, seafood, fortified wheat-based cereals	Meat, poultry, fortified cereals, fortified alternative beverages	2–6 years: 1.5 μg 7–10 years: 2.5 μg 11–13 years: 3.5 μg	Serum vitamin B12 and serum folate level [105, 106]
**Riboflavin**	Milk and dairy products, fortified wheat-based cereals, eggs	Meat, fortified alternative beverages, fortified cereals	2–3 years: 0.5 mg 4–8 years: 0.6 mg 9–13 years: 0.9 mg	Serum riboflavin level [107]

* Türkiye Dietary Guidelines-2016 [51].

**Table 3:** Range of nutrients content of milk substitutes (per 100 mL).

	Cow’s milk*	AAF	HRF	Soy-BB	Almond-BB	Hazelnut-BB	Coconut-BB	Oat-BB
Energy (kcal)	60–64	64–100	67–69	30–61	19–34	29–42	20–34	43–58
Carbohydrate (g)	4.4–5.7	6.62–11.8	7–7.6	2.5–7.8	0.6–4.1	2.2–5.9	2.7–2.8	5.1–7
Protein (g)	2.9–3.6	1.8–2.8	1.6–1.9	2–3.1	0.5–0.7	0.4–0.6	0.1–0.3	0.3–0.6
Fat (g)	3.2–4	3.4–4.6	3.4	1.3–1.8	1.1–1.9	1.6–2.1	0.9–2.4	1.3–3.0
Iron	0.01–0.03	1–1.22	0.7–1.1	N/A	N/A	N/A	N/A	N/A
Calcium (mg)	70–116	65.6–90.3	74–82	120**	120**	120**	120**	120**
Vitamin D (mcg)	0–0.5	1–1.3	1.5	0.75**	0.75**	0.75**	0.75**	1.0**
Vitamin B2 (mg)	0.17–0.22	0.07–0.2	0.15–0.16	0.21**	0.21**	0.21**	N/A	(0.2–0.21)**
Vitamin B12 (mcg)	0.4–0.47	0.18–0.25	0.2	0.38**	(0.38–0.4)**	0.38**	(0.38–0.4)**	(0.38–0.4)**
Vitamin E (mg)	0.1–0.16	0.67–1.4	1.2–1.3	N/A	1.8**	1.8**	1.8**	1.8**

AAF: Amino acid-based formula, HRF: Hydrolyzed rice-based formula, BB: Based beverage

* Whole milk without added vitamin D (Türkkomp)

** In case of fortification,

N/A: Not available
